# The seven enigmas of SARS-CoV-2: From the past to the future

**DOI:** 10.70962/jhi.20250149

**Published:** 2025-10-24

**Authors:** Evangelos Andreakos, Lisa Arkin, Paul Bastard, Alexandre Bolze, Alessandro Borghesi, Petter Brodin, Jean-Laurent Casanova, Giorgio Casari, Aurélie Cobat, Beth Drolet, Jacques Fellay, Elena W.Y. Hsieh, Isabelle Meyts, Trine H. Mogensen, Vanessa Sancho-Shimizu, András N. Spaan, Helen C. Su, Donald C. Vinh, Ahmad Yatim, Qian Zhang, Shen-Ying Zhang, COVID Human Genetic Effort, Laurent Abel, Alessandro Aiuti, Saleh Al-Muhsen, Evangelos Andreakos, Andrés A. Arias, Lisa M. Arkin, Hagit Baris Feldman, Paul Bastard, Alexandre Bolze, Anastasiia Bondarenko, Alessandro Borghesi, Ahmed A. Bousfiha, Petter Brodin, Giorgio Casari, John Christodoulou, Aurélie Cobat, Roger Colobran, Antonio Condino-Neto, Stefan N. Constantinescu, Beth A. Drolet, Munis Dündar, Sara Espinosa-Padilla, Jacques Fellay, Carlos Flores, Antoine Froidure, Guy Gorochov, David Hagin, Rabih Halwani, Lennart Hammarström, Elena W.Y. Hsieh, Yuval Itan, Emmanuelle Jouanguy, Elżbieta Kaja, Yu-Lung Lau, Davood Mansouri, László Maródi, Isabelle Meyts, Trine H. Mogensen, Lisa F.P. Ng, Antonio Novelli, Giuseppe Novelli, Satoshi Okada, Keisuke Okamoto, Firat Ozcelik, Qiang Pan-Hammarström, Rebeca Perez de Diego, David S. Perlin, Anne Puel, Aurora Pujol, Laurent Renia, Vanessa Sancho-Shimizu, Mohammad Shahrooei, Anna Shcherbina, Ondrej Slaby, Pere Soler-Palacín, András N. Spaan, Ivan Tancevski, Stuart G. Tangye, Ahmad Abou Tayoun, Christian Thorball, Pierre Tiberghien, Stuart E. Turvey, Donald C. Vinh, Qian Zhang, Shen-Ying Zhang, Helen C. Su, Jean-Laurent Casanova

**Affiliations:** 1 Laboratory of Immunobiology, Center for Clinical Research, Experimental Surgery and Translational Research, Biomedical Research Foundation of the Academy of Athens, Athens, Greece; 2 Department of Dermatology, University of Wisconsin School of Medicine and Public Health, Madison, WI, USA; 3 Laboratory of Human Genetics of Infectious Diseases, Necker Branch, Institut National de la Santé et de la Recherche Médicale (INSERM) U1163, Necker Hospital for Sick Children, Paris, France; 4 Imagine Institute, Paris Cité University, Paris, France; 5 St. Giles Laboratory of Human Genetics of Infectious Diseases, Rockefeller Branch, Rockefeller University, New York, NY, USA; 6 Pediatric Hematology-Immunology and Rheumatology Unit, Necker Hospital for Sick Children, Assistance Publique-Hôpitaux de Paris, Paris, France; 7 Helix, San Mateo, CA, USA; 8 Host-Pathogen Group and Neonatal Intensive Care Unit, 'IRCCS' San Matteo Research Hospital, Pavia, Italy; 9 Department of Women’s and Children’s Health, Karolinska Institutet, Stockholm, Sweden; 10 Stockholm Department of Immunology and Inflammation, Imperial College London, London, UK; 11 Medical Research Council Laboratory of Medical Sciences, Imperial College Hammersmith Campus, London, UK; 12 Howard Hughes Medical Institute, New York, NY, USA; 13 Department of Pediatrics, Necker Hospital for Sick Children, Paris, France; 14 Vita-Salute San Raffaele University, Milan, Italy; 15 IRCCS San Raffaele Scientific Institute, Milan, Italy; 16 School of Life Sciences, École Polytechnique Fédérale de Lausanne, Lausanne, Switzerland; 17 Biomedical Data Science Center, Lausanne University Hospital and University of Lausanne, Lausanne, Switzerland; 18 Department of Pediatrics, Section of Allergy and Immunology, Department of Immunology and Microbiology, University of Colorado School of Medicine, Aurora, CO, USA; 19 Laboratory for Inborn Errors of Immunity, Department of Microbiology, Immunology and Transplantation, KU Leuven, Leuven, Belgium; 20 Department of Pediatrics, University Hospitals Leuven, Leuven, Belgium; 21 Department of Biomedicine, Aarhus University, Aarhus, Denmark; 22 Department of Infectious Diseases, Aarhus University Hospital, Aarhus, Denmark; 23 Department of Infectious Disease, Faculty of Medicine, Imperial College London, London, UK; 24 Centre for Paediatrics and Child Health, Faculty of Medicine, Imperial College London, London, UK; 25 Department of Medical Microbiology, University Medical Center Utrecht, Utrecht University, Utrecht, Netherlands; 26 Human Immunological Diseases Section, Laboratory of Clinical Immunology and Microbiology, Intramural Research Program, National Institute of Allergy and Infectious Diseases, National Institutes of Health, Bethesda, MD, USA; 27 Department of Medicine, Division of Infectious Diseases, McGill University Health Centre, Montréal, Canada; 28 Centre of Reference for Genetic Research in Infection and Immunity, Research Institute-McGill University Health Centre, Montréal, Canada; 29 Neonatal Intensive Care Unit, Mother, Child and Adolescent Department, Geneva University Hospitals, Geneva, Switzerland; 30 Department of Pediatrics, Gynecology and Obstetrics, University of Geneva, Geneva, Switzerland; 31 School of Life Sciences, Swiss Federal Institute of Technology, Lausanne, Switzerland

## Abstract

Five years ago, we launched the COVID Human Genetic Effort. Our goal was to explain the clinical variability among SARS-CoV-2–exposed individuals by searching for monogenic inborn errors of immunity and their phenocopies. We deciphered the pathogenesis of critical COVID-19 pneumonia and multisystem inflammatory syndrome in children in ∼15% and 2% of cases, respectively, thereby revealing general mechanisms of severe disease. We also defined neuro-COVID genetically and immunologically in one child, while we delineated the immunological mechanisms of COVID-toes in healthy children and young adults, paving the way for their genetic study. Understanding the human genetic and immunological basis of resistance to SARS-CoV-2 infection, long COVID, and myocarditis after mRNA vaccination has been challenging, and investigations remain ongoing. This work highlights the power of patient-based basic research and large-scale international collaborative efforts to discover human genetic and immunological drivers of infectious disease phenotypes, with implications for the timely development of new medical strategies before the next pandemic arrives.

## Teaser

Andreakos et al. report the progress of the COVID Human Genetic Effort since its launch 5 years ago, with the aim to understand clinical variability, from resistance to severe outcomes, among individuals exposed to SARS-CoV-2. Their work revealed key mechanisms of pneumonia, MIS-C, and other manifestations, informing diagnostics, therapeutics, and preparedness for future pandemics.

## Graphical Abstract

**Figure:**
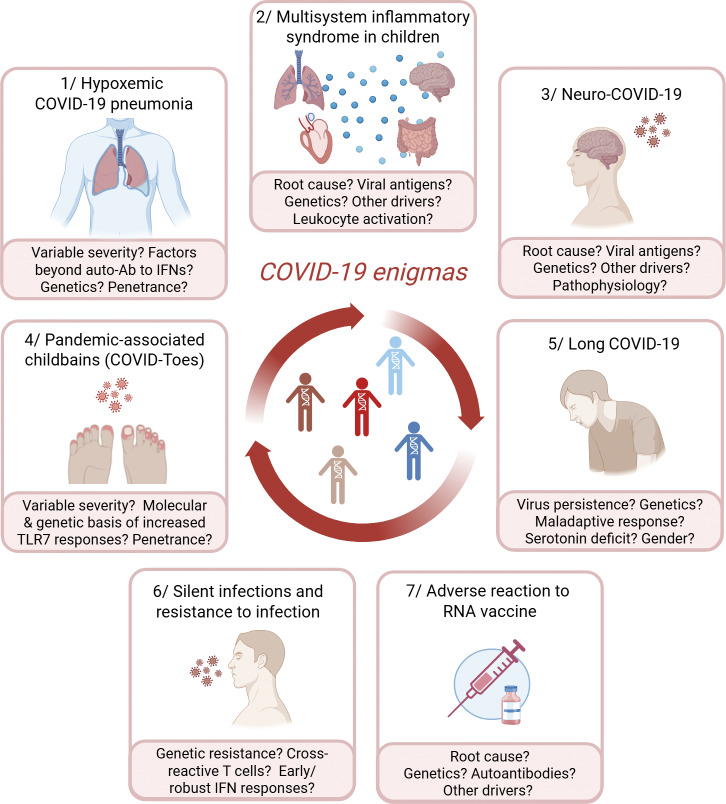

## Introduction

**Figure 1.**
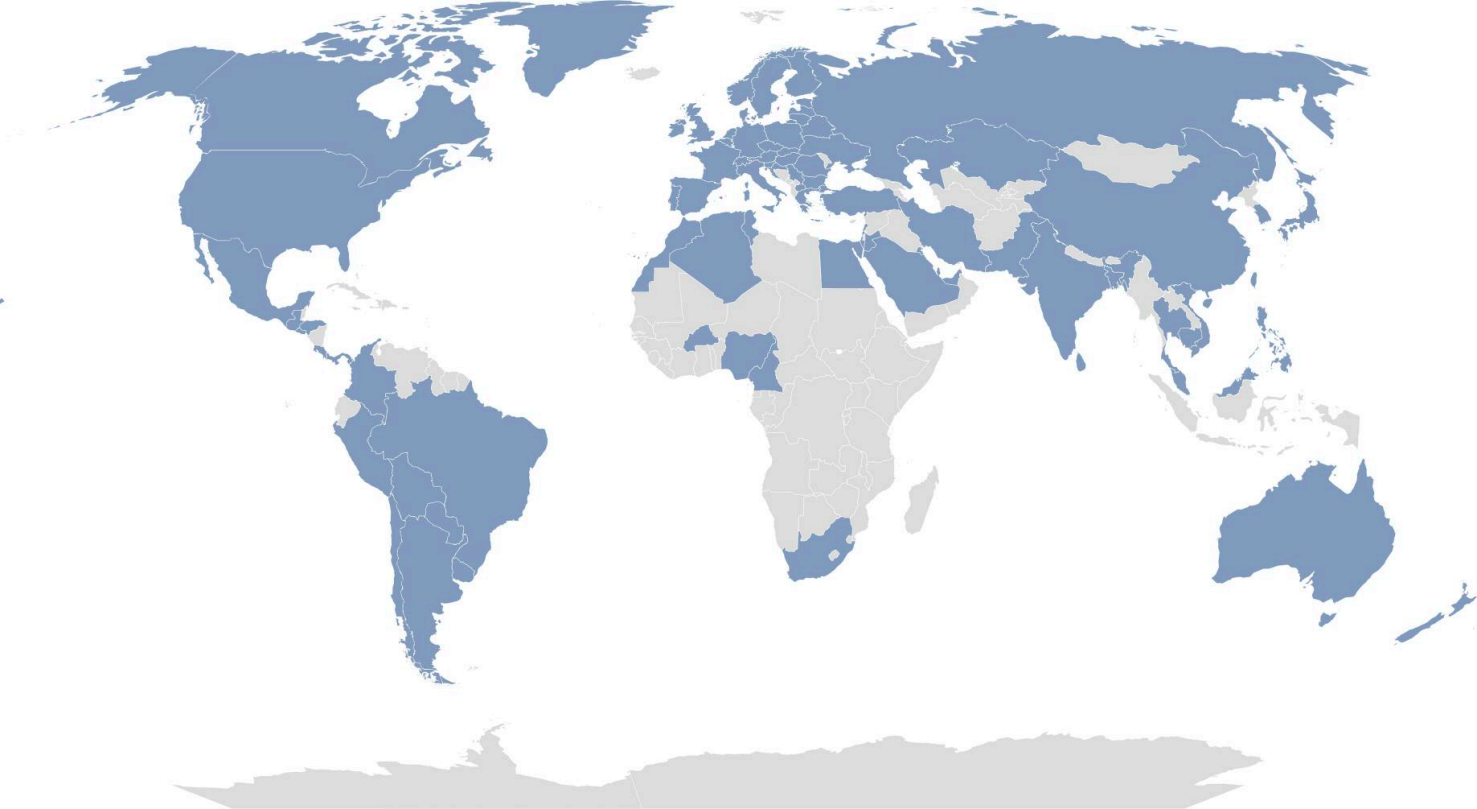
**CHGE world map.** Our collaborators represent the following 85 countries: Algeria, Argentina, Australia, Austria, Bangladesh, Belarus, Belgium, Bolivia, Brazil, Bulgaria, Burkina Faso, Cambodia, Cameroon, Canada, Chile, China, Colombia, Costa Rica, Croatia, Czech Republic, Denmark, Egypt, El Salvador, Estonia, Finland, France, Germany, Greece, Greenland, Guatemala, Honduras, Hong Kong, Hungary, India, Iran, Israel, Italy, Japan, Jordan, Kazakhstan, Kuwait, Latvia, Lebanon, Lithuania, Malaysia, Malta, Mexico, Morocco, Netherlands, New Zealand, Nigeria, North Macedonia, Norway, Pakistan, Panama, Paraguay, Peru, Philippines, Poland, Portugal, Qatar, Republic of Ireland, Romania, Russia, Saudi Arabia, Serbia, Singapore, Slovakia, Slovenia, South Africa, South Korea, Spain, Sri Lanka, Sweden, Switzerland, Taiwan, Thailand, Tunisia, Turkey, Ukraine, United Arab Emirates, the United Kingdom, the United States, Uruguay, and Vietnam.

The SARS-CoV-2 pandemic provided the unique opportunity to study the human genetic and immunological determinants underlying a great diversity of clinical manifestations: in some individuals, the new virus caused acute, life-threatening manifestations, while in most individuals, the virus provoked only silent or benign manifestations. We launched the COVID Human Genetic Effort (CHGE) in February 2020 to try to tackle this global public health problem by means of forward genetics, searching for monogenic inborn errors of immunity (IEI), or their autoimmune or somatic phenocopies, in patients with critical COVID-19 pneumonia (https://www.covidhge.com/) (Fig. 1). Our hypothesis was that the virus is not by itself causal of disease, nor of any specific clinical manifestations, but acts as an environmental trigger in predisposed individuals, thereby revealing human genetic and immunological causes and mechanisms of disease (1).

Other consortia have applied genome-wide association study (GWAS) approaches to COVID-19, assuming that common variants in multiple genes, each exerting small effect sizes, work together in combination to increase disease risk in any given individual (2). However, the CHGE has taken an alternative approach based upon our collective experience that a rare variant can exert large effect sizes to cause extreme disease outcomes in a given individual (3). Our strategy has been successful because we have recruited patients with outlier presentations, defined stringently by objective criteria, while also recruiting internationally to expand sample sizes of these rare presentations, thereby overcoming genetic heterogeneity.

Previous human monogenic studies of viral diseases had focused on endemic and seasonal infections, such as herpes simplex encephalitis and influenza pneumonia, respectively (3, 4). With the SARS-CoV-2 pandemic, we applied a similar forward and reverse genetics approach, at the global population level. A major factor contributing to our success was that we studied a primary viral infection, against which everyone was naïve, at a massive scale within a few months. Our approach led to the identification of the pathogenesis of critical COVID-19 pneumonia in an estimated 15–20% of cases. The identification of inborn errors of and autoantibodies to type I interferons (IFNs) in these patients even provided a general mechanism of disease (5, 6).

**Figure 2.**
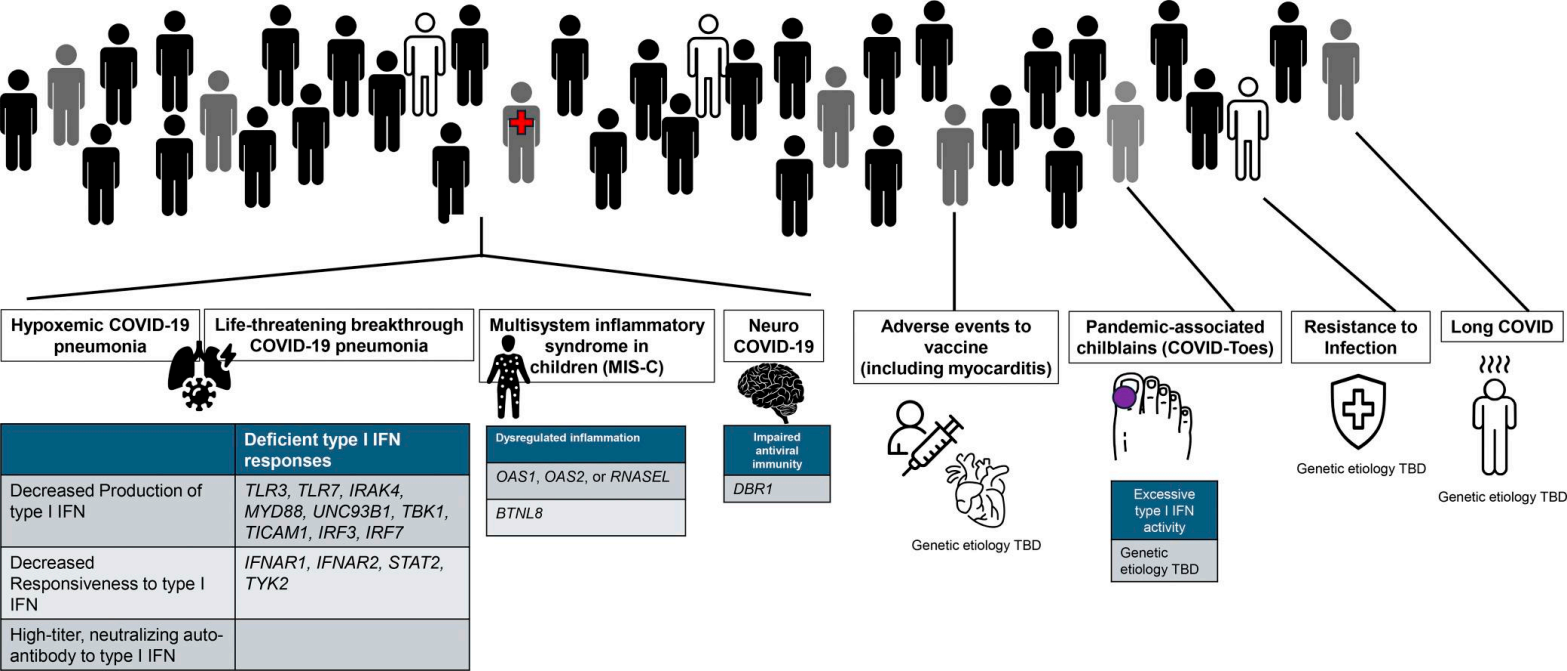
**Etiologies are unknown for four of the seven enigmas: resistance to infection (and silent infection), pandemic chilblains (although a mechanism of disease is documented), long COVID, and mRNA vaccine–induced myocarditis.** There are etiologies for the other three enigmas. Hypoxemic COVID-19 pneumonia is explained in 15–20% of cases by inborn errors of or autoantibodies against type I IFNs. MIS-C is explained in 1–2% of cases by other inborn errors. Only one patient with neuro-COVID is understood etiologically.

While we initially focused our studies on (1) hypoxemic critical pneumonia and (2) multisystem inflammatory syndrome in children (MIS-C), our early discoveries facilitated expansion of our search to include other phenotypes: (3) viral encephalitis and acute inflammation of the central or peripheral nervous system (neuro-COVID), (4) virus-triggered chilblains (COVID-toes), (5) long COVID, and (6) resistance to infection in highly exposed individuals. When mRNA vaccines became available, we added a seventh enigma, postvaccine myocarditis (Fig. 2). For each problem, we followed the same approach, searching for single-gene IEI, while characterizing the clinical and immunological features of the corresponding patients, thereby deciphering genetic causes and immunological mechanisms at the molecular and cellular levels. Below we review the seven medical enigmas in detail, including the advances we have made and ongoing studies.

## Seven medical enigmas

### 1. Hypoxemic COVID-19 pneumonia

The biggest enigma surrounding SARS-CoV-2 is the variable severity of respiratory infections among infected individuals, which ranges from asymptomatic to life-threatening. Based upon prepandemic work demonstrating that monogenic defects can underlie a narrow susceptibility to other respiratory viral diseases, we hypothesized that monogenic defects might also underlie life-threatening COVID-19 pneumonia (1). Our first breakthrough was the discovery of inborn errors in TLR3-dependent type I IFN production and response in patients with hypoxemic COVID-19 pneumonia, which showed that critical COVID-19 and influenza pneumonia can indeed be allelic (7). Thereafter, we and others identified X-linked recessive TLR7 deficiency, which underlies hypoxemic COVID-19 pneumonia in males (8, 9, 10, 11, 12). Inborn errors in 13 genes, which either induce type I IFNs (*TLR3*, *TLR7*, *IRAK4*, *MYD88*, *UNC93B1*, *TBK1*, *TICAM1*, *IRF3*, *IRF7*) or govern responses to type I IFNs (*IFNAR1*, *IFNAR2*, *STAT2*, *TYK2*), were subsequently demonstrated to underlie critical COVID-19 pneumonia, often in young or middle-aged patients (2, 7, 9, 13, 14, 15, 16, 17). The inborn errors identified in these patients are rare, except for a dominant negative, hypomorphic IFNAR1 variant (p.Pro335del), which is common in Southern China (minor allele frequency [MAF]∼2%) (13, 18, 19, 20).

Strikingly, we discovered autoantibodies (mostly IgG) neutralizing type I IFNs in 15% of patients with critical COVID-19 pneumonia and 20% of patients who died from COVID-19 (21, 22, 23, 24, 25). These autoantibodies preexist infection and are common in the general population sampled prior to 2019 (0.2–1%), where they increase with age, reaching almost 7% in people over 80 years old (22). Together, these two parallel and complementary discoveries indicated that human type I IFN immunity is indispensable for defense against SARS-CoV-2 infection in the lung (6). Multiple large-scale GWAS have identified over 50 genomic regions containing common variants that are associated with COVID-19 severity with modest effect sizes (26, 27, 28, 29). The first reported and strongest signal (odds ratio [OR] of 1.6) for severe pneumonia came from a haplotype inherited from Neanderthals (2, 30). Interestingly, seven loci seemingly associated with COVID-19 severity were possibly linked to the type I IFN pathway, *IFNAR2*, *TYK2*, *JAK1*, *OAS1*, *IRF1*, *IFNA10*, and *DOCK2* (2, 26, 29).

Genetic defects that underlie COVID-19 pneumonia, as well as auto-Ab-IFN, also underlie other severe viral diseases. These include encephalitis triggered by herpes simplex virus, Japanese encephalitis virus, tick-borne encephalitis virus, West Nile virus (WNV), or enterovirus (*TLR3*, *UNC93B1*, *TBK1*, *TICAM1*, *IRF3*, *IFNAR1*, and auto-Ab-IFN) (20, 31, 32, 33, 34, 35, 36, 37, 38); influenza pneumonia (*TLR3*, *IRF7*, *STAT2*, and auto-Ab-IFN) (15, 39, 40, 41); adverse reactions to live-attenuated viral vaccines (*IFNAR1*, *IFNAR2*, *STAT2*, and auto-Ab-IFN) (18, 19, 20, 42, 43, 44); and Middle East respiratory syndrome pneumonia (auto-Ab-IFN) (45). Incomplete penetrance for any given viral disease is apparently common in patients with defective type I IFN immunity, as almost all patients reported only had an isolated, single episode of severe infection, despite their previous exposure to several of the above-listed viruses. Incomplete penetrance and variable expressivity may result from different levels of molecular redundancies, including contributions by other sensors such as MDA5 and RIG-I, which like TLR3 and TLR7 are also capable of sensing the same viruses; the type III IFN pathway, whose downstream antiviral responses overlap with the type I IFN pathway; or adaptive immunity including virus-specific or cross-reactive antibody responses due to prior viral exposures or vaccination. Mutations in IFN-stimulated genes (ISGs) may reveal more specific phenotypes than mutations in IFN-inducing genes or the core IFN pathway. These findings may also help stratify the patients who will most benefit from IFN therapy for COVID or other viral diseases.

### 2. MIS-C

MIS-C is an inflammatory disease triggered by SARS-CoV-2 infection that, for unknown reasons, predominantly occurs in children, although it has very rarely been reported in adults (referred to as MIS-A) (46, 47, 48). MIS-C typically occurs 4 wk after benign infection. When it initially emerged in 2020, it affected children with a median age of 8 to 9 years, and had an estimated prevalence of about 1–2 per 10,000 infected children (49, 50, 51, 52). The dramatic decrease in the number of MIS-C cases since mid-2022 suggests that both vaccine and previous infection protect from MIS-C (53, 54). The new viral strains themselves may be less pathogenic in unvaccinated children (55, 56). Children with MIS-C presented with fever, rash, abdominal pain, myocarditis, and other clinical features reminiscent of a classic pediatric inflammatory condition, Kawasaki disease (KD), including lymphadenopathy, coronary aneurysm, and elevated markers of inflammation (48, 57, 58, 59, 60, 61); hence, initial reports described MIS-C as an atypical form of KD. Blood markers of cardiovascular endothelial injury (troponin, B-type natriuretic peptide) and gastrointestinal epithelial injury (LPS-binding protein, soluble CD14) are common (62). Various leukocyte subsets are also affected, including sustained monocyte activation as suggested by the high levels of proinflammatory markers, including ferritin, IL-6, IL-10, IL-18, MCP1 (CCL2), IL-1RA, and TNF, as well as neutrophilia. In addition, increased type II IFN (IFN-γ) signaling, not necessarily specific to monocyte activation, is prevalent during the early phase of disease (58, 62, 63, 64, 65, 66, 67). Finally, a unique immunological phenotype in MIS-C (detected in up to ∼75% of patients) involves a polyclonal expansion of Vβ21.3 on CD4^+^ and CD8^+^ T cells (62, 64, 66, 68, 69), suggestive of a viral superantigen driving a specific activation and expansion of T cells. In this multitude of molecular and cellular abnormalities, the root cause of MIS-C was enigmatic (70).

Searching for monogenic IEI underlying MIS-C via an unbiased genome-wide approach, in 2023 the CHGE reported the surprising finding of autosomal recessive deficiencies of the OAS-RNase L pathway in five children with biallelic mutations in *OAS1*, *OAS2*, or *RNASEL*, representing ∼1% of the large international cohort studied (71). The type I IFN–inducible dsRNA-sensing proteins OAS1 and OAS2 normally generate 2′-5′-linked oligoadenylates (2–5A) to activate the single-stranded RNA (ssRNA)-degrading RNase L, which is particularly active in mononuclear phagocytes. The OAS-RNase L pathway has been studied for years as an antiviral pathway in vitro and in mice in vivo (72, 73, 74, 75). However, in humans under natural conditions of infection by SARS-CoV-2, deficiency of this pathway resulted in acute postinfectious systemic hyperinflammation. Monocytic cell lines, primary monocytes, and monocyte-derived dendritic cells with genetic deficiencies of OAS1, OAS2, or RNase L produce excessive amounts of inflammatory cytokines in response to intracellular dsRNA or SARS-CoV-2. Conversely, and consistent with the lack of pneumonia in patients with MIS-C, a lung epithelial cell line and fibroblasts defective for this pathway restrict SARS-CoV-2 normally, unlike IFNAR1-deficient cells from patients prone to hypoxemic pneumonia without MIS-C. Single-gene recessive inborn errors of the OAS-RNase L pathway unleash the production of SARS-CoV-2–triggered inflammatory cytokines by mononuclear phagocytes, thereby underlying MIS-C. These findings provide mechanistic underpinnings to the clinically driven treatment algorithms for MIS-C, and may further refine current guidelines as additional discoveries are made.

Subsequently, another study using a gene burden analysis identified enrichment of rare variants in *BTNL8*, a gene regulating intestinal γδT cells, in MIS-C patients (76). Approximately 2% of the MIS-C cohort tested carried rare predicted deleterious variants of *BTNL8*. These variants contributed to a fourfold increased odds of presenting with MIS-C. The prominent site of *BTNL8* expression is the gut epithelium, where it regulates Vγ4^+^ γδT cells critical for gut barrier integrity. Given the known association of *BTNL8* with inflammatory bowel disease severity, it is believed that BTNL8 may play a role in regulating intestinal inflammation seen in MIS-C. Although intestinal tissue from MIS-C patients was unavailable, the association between *BTNL8* variants and serum markers of increased gut permeability was noted. BTNL8 is thus thought to regulate intestinal inflammation upon SARS-CoV-2 infection, such that those with impaired *BTNL8* function are unable to resolve intestinal inflammation that subsequently leads to systemic inflammation. Overall, discoveries of IEI in MIS-C have highlighted unchecked mononuclear phagocyte-driven inflammation (OAS-RNase L pathway) and intestinal inflammation (BTNL8) as key features of MIS-C pathogenesis. Many questions remain, including those surrounding the exact SARS-CoV-2–related RNA products triggering monocyte activation and the clonal activation and expansion of Vβ21.3^+^ T cells. A recent study suggested that EBV reactivation contributes to the T cell expansion in MIS-C (77). Further investigation is warranted for the remaining majority of patients whose genetic and immunological cause of MIS-C remains unknown. MIS-A remains entirely enigmatic; it may represent a forme fruste of MIS-C in some patients, and a distinct hyperinflammatory syndrome with unique molecular drivers in others. Other pediatric inflammatory disorders like KD may share similar pathogenic mechanisms with MIS-C, which could be clarified using similar monogenic investigatory approaches.

### 3. Neuro-COVID

SARS-CoV-2 is primarily a respiratory virus, but various neurological manifestations have been observed in infected individuals, either accompanying acute COVID-19 pneumonia or presenting as isolated neurological conditions. Previous papers describing “neuro-COVID” mostly focused on the description of different neurological illnesses, attesting to the neuroinvasiveness of the virus, or consequences of SARS-CoV-2–triggered cytokine storm and systemic inflammation (78, 79). These SARS-CoV-2–related neurological conditions can be grouped into those affecting the central nervous system (CNS), including meningoencephalitis, encephalopathy, and acute demyelinating encephalomyelitis, and those affecting the peripheral nervous system in the form of the acute flaccid paralysis of Guillain–Barré syndrome (GBS) or various myopathies (80, 81). Neuro-COVID may also be categorized depending on the temporal association with SARS-CoV-2 infection into acute, subacute, or long-term neurological presentations, such as encephalitis, GBS, or long COVID, respectively (78). Moreover, COVID-19–associated coagulopathy, cerebral vasculopathy, and microthrombosis may also lead to brain inflammation and pathology manifesting as neurological symptoms and stroke (78), which is not considered within the group of neuro-COVID described here.

Intriguingly, SARS-CoV-2 has been only rarely identified in the cerebrospinal fluid during acute COVID-19 or by postmortem pathology examination of brain tissue from humans succumbing to COVID-19, leaving it unclear to what extent the virus productively replicates in neurons or other cells of the CNS in vivo (79, 82). CNS invasion has been suggested to occur through viral entry at the level of the olfactory bulb, the vascular endothelium, and choroid plexus, or by migration of SARS-CoV-2–infected monocytic cells across the blood–brain barrier like a Trojan horse (83). However, SARS-CoV-2 RNA and nucleocapsid protein have been demonstrated in human neural progenitor cells and human brain organoids infected in vitro, suggesting SARS-CoV-2 permissiveness and productive replication in these cells (82, 84). In contrast, postmortem brain histopathology sections from patients revealed meningeal inflammation, neuronal loss, and hypoxia/ischemia as prominent features, with only low levels of virus detectable and in only a minority of cases (79, 82). Despite the various neurological clinical presentations suggesting CNS involvement, and numerous studies on single patients or small groups of patients, key aspects of SARS-CoV-2 neuroinvasion, neurotropism, and neuropathogenesis remain poorly understood (79, 82, 83, 84).

The research of human monogenic etiologies of severe infection was previously successful at dissecting the pathogenesis of isolated viral encephalitis, especially triggered by herpes simplex virus 1 (HSV-1), but also other viruses including varicella zoster virus and influenza virus (70, 85). Taking this same approach, a study reported a recessive DBR1 defect underlying isolated SARS-CoV-2 brainstem encephalitis in a 14-year-old boy of Syrian origin (86). The patient’s magnetic resonance imaging showed encephalitic lesions in the pons, mesencephalon, and cerebellum, and evidence of increased intracranial pressure. Genetic evaluation revealed that the patient was homozygous for a loss-of-function variant in the RNA debranching enzyme DBR1, a hypomorphic and pathogenic variant previously reported to underlie brainstem encephalitis by HSV-1 in other patients (87). Fibroblasts from the patient showed low levels of DBR1 and accumulation of RNA lariats. Moreover, human induced pluripotent stem cell (hPSC)–derived hindbrain neurons with this DBR1 genotype were highly susceptible to SARS-CoV-2 infection. Likewise, neurons derived from healthy control hPSCs and exogenously challenged with RNA lariats showed increased viral susceptibility. Finally, reconstitution of wild-type DBR1 expression in patient fibroblasts and hPSC-derived hindbrain neurons rescued the RNA lariat accumulation phenotype. This paper is the first account of a distinctive IEI associated with a neuro-COVID phenotype, in this case, rare acute brainstem encephalitis. Future search for single-gene IEI underlying encephalitis and other neuro-COVID phenotypes will clarify the pathophysiological principles of neurological disease manifestations triggered by SARS-CoV-2. Such studies are likely to also provide insights relevant to other neuroinfections with human pathogenic neurotropic viruses.

### 4. Pandemic-associated chilblains (COVID-toes)

The sudden and unprecedented global outbreak of chilblain lesions, which paralleled early waves of SARS-CoV-2 infections, long remained unexplained. Chilblains are tender, inflammatory papules that appear on acral sites. Although first described in 1888 (88), only recent evidence has linked some cases to excessive type I IFN responses. Chilblains can occur in the context of chronic type I IFN–driven disorders such as the monogenic interferonopathies (89, 90, 91) and systemic lupus erythematosus (88, 92, 93). However, when affecting otherwise healthy individuals, they are considered primary and idiopathic. Before the onset of the pandemic, primary forms were rare (94) and typically occurred in clusters during winter months (95, 96, 97), coinciding with increased circulation of ssRNA respiratory viruses such as common coronaviruses, RSV, and influenza virus (98), when the ambient temperature was colder. However, a direct connection to viral triggers had neither been firmly established nor strongly suspected before 2020.

During the early months of the COVID-19 pandemic, the surge in chilblain cases among otherwise healthy, relatively young individuals, known as pandemic-associated chilblains (PC) or so-called “COVID-toes,” garnered considerable scientific and public interest (94, 99, 100, 101, 102). The temporal clustering of PC and COVID-19 cases suggested a causal link between SARS-CoV-2 and chilblains (103, 104, 105, 106, 107). However, most patients tested negative for SARS-CoV-2 by nasopharyngeal PCR at the time of chilblain onset and lacked detectable adaptive responses (overall IgG seroconversion <10%) (108, 109, 110, 111, 112). While some authors attributed PC outbreaks to behavioral changes during lockdown (109, 113, 114), accumulating evidence supports the hypothesis of an innate immune phenomenon triggered by early SARS-CoV-2 variants (115, 116, 117, 118, 119, 120). Epidemiologically, PC onset was correlated with SARS-CoV-2 circulation (94, 97) and linked to household exposure (121, 122). Virologically, evidence of an abortive SARS-CoV-2 infection was supported by detection of SARS-CoV-2 RNA debris in PC samples without ongoing viral replication (123). Immunologically, patients with PC displayed high levels of systemic and local type I IFN activity (117, 120, 123, 124), often accompanied by transient IgA responses but rarely IgG seroconversion (117, 120, 125), suggesting viral exposure and a robust innate antiviral activation with limited adaptive priming.

Consistent with the essential role of type I IFN in protective immunity against SARS-CoV-2 (7, 9, 21, 22) and the deleterious effects of excessive type I IFN activity (126), a leading mechanistic hypothesis posits that individuals with PC are primed to mount an enhanced type I IFN response to SARS-CoV-2, promoting early viral clearance and the subsequent development of chilblains. Indeed, patients’ leukocytes produce abnormally high levels of IFN-I when stimulated with ssRNA viruses, especially SARS-CoV-2, but not DNA viruses (124). Moreover, patients’ plasmacytoid dendritic cells (pDCs) display cell-intrinsic hyperresponsiveness to TLR7 stimulation (124), in contrast to pDCs from TLR7-deficient patients who are prone to critical COVID-19 pneumonia. Chilblains result from the infiltration of activated pDCs in acral skin (123, 124), with type I IFN–mediated inflammation at acral sites, a response likely augmented by cold-induced vasoconstriction and endothelial damage. Hence, PC might signal enhanced TLR7 activity, which confers strong—often sterilizing—innate immunity to SARS-CoV-2 infection, limiting antigen availability for adaptive priming. In the aggregate, these results offer a mechanistic explanation for both strong temporal association of primary chilblains during the early pandemic and the absence of conventional markers of adaptive responses in affected individuals.

The COVID-19 pandemic provided an opportunity to revisit the pathogenesis of primary chilblains, challenging the conventional belief that “idiopathic” chilblains result solely from exposure to cold, and suggesting that chilblains may be primarily triggered by immune responses to ssRNA viruses, developing as a trade-off from excessive but transient type I IFN responses in predisposed individuals. The molecular and genetic basis of enhanced TLR7 responses to SARS-CoV-2 in patients with chilblains has yet to be elucidated, although TLR7 hyperactivation is known to induce other autoimmune disorders including lupus, of which chilblains are a common cutaneous manifestation (127).

Additional viruses that could also trigger primary chilblains remain to be discovered. The observation of multiple family members developing PC simultaneously supports a genetic predisposition with a dominant inheritance pattern (100, 118, 128). Through enrollment of a dedicated cohort with chilblains, the CHGE continues to investigate whether these affected individuals carry specific genetic variants that enhance TLR7-mediated immunity to SARS-CoV-2 and other ssRNA viruses.

### 5. Long COVID

The severity of acute infections varies as a consequence of host genetics and immune function with different manifestations across age groups (129). Postinfectious diseases represent another form of pathology triggered by infections in individuals with likely predisposing genetic and environmental characteristics. Postinfectious syndromes have previously been reported following infections with dengue viruses (130), Ebola virus (131), chikungunya (132), WNV (133), Epstein-Barr virus (134), influenza (135), and SARS (136), but mechanisms of these diseases are poorly understood. Genetic etiologies have not been reported. SARS-CoV-2 infections and their associated postinfectious disease, long COVID, offer a unique opportunity for unraveling the pathogenic process and possible shared mechanisms with other postinfectious diseases given the global spread, across diverse populations with a novel virus. The diagnostic criteria for long COVID are vague, and consequently, the incidence rates vary widely. To mitigate this uncertainty, we have decided to focus on the most severe cases with objective measures of disease (137). The most frequent symptoms are fatigue, dysautonomia, cognitive impairments, microvascular dysfunction, and fevers with minimal evidence of systemic inflammation using clinical chemistry measurements (137).

Multiple lines of evidence point toward viral persistence, suggestive of chronic infection, in patients with long COVID. In some individuals, viral antigens (138) and antisense RNA (139) are measurable in plasma, while most patients show elevated levels of SARS-CoV-2–specific antibodies (140, *Preprint*) and SARS-CoV-2–specific B cells undergoing somatic hypermutation for many months following initial SARS-CoV-2 infection (141). SARS-CoV-2–specific T cells have been described as exhausted, while our own data indicate a restrained expansion of such cells (140, *Preprint*). The latter suggests maladaptive disease tolerance as an underlying mechanism of disease, similar to previous results in the related condition, myalgic encephalomyelitis (142). Striking sex differences in incidence in postinfectious diseases are seen in general and long COVID in particular, with 75–80% of individuals being females of reproductive age (140, *Preprint*). Hence, we speculate that sex differences in immune system composition and function, including the role of sex hormones as dynamic modulators of female-specific traits during reproductive age, may be important for development of long COVID (143). It is intriguing that women with long COVID have autoantibodies to nonimmune targets (144, *Preprint*). While this provides a glimpse into immunological aberrancies in some persons living with long COVID, the underlying molecular and genetic bases remain enigmatic.

Beyond the significant role of the immune system in long COVID, both during and after the acute phase, emerging evidence suggests that disruption of the serotonin system might contribute to the development of post–SARS-CoV-2 sequelae (145). The serotonin system may be linked to type I IFN activity, which could contribute to serotonin depletion through several mechanisms: (1) reducing tryptophan uptake, the precursor to serotonin, via inflammation in the gut, (2) hyperactivating platelets and diminishing their serotonin storage, and (3) increasing serotonin breakdown via monoamine oxidase. Studies of serotonin-related phenotypes in patients with IEI of type I IFN are warranted. Collectively, these processes result in peripheral serotonin deficiency, which impairs vagal signaling crucial for cognitive functions, especially memory. This deficiency also contributes to chronic inflammation, hypercoagulability, and autonomic dysfunction.

Given the highly variable presentation and severity of long COVID cases, the CHGE is focusing on outlier phenotypes, similar to our approach taken for hypoxemia pneumonia and MIS-C. Using measures of disease such as autonomic dysfunction, microvascular dysfunction, and/or persistent viral antigens, we have already enrolled over 300 patients with the most severe forms of long COVID and objective markers of disease for human genetic studies. We will test models of genetic homogeneity and heterogeneity, focusing on rare genotypes.

### 6. Silent infections and resistance to infection

After exposure to SARS-CoV-2, many individuals remain clinically asymptomatic. Some of these remain PCR test–negative and seronegative and appear to be resistant to infection. “Silent or unapparent infection” is a long-standing observation by Charles Nicolle during the “Golden Age of Microbiology.” This phenomenon is seen with innumerable infections, but despite advances in understanding susceptibility to severe disease, resistance to infection or asymptomatic infection remains largely enigmatic. Among individuals who demonstrate positive PCR tests and/or seroconversion, up to 40% remain clinically silent (146, 147, 148). In many of these individuals, robust T cell responses develop, despite a complete lack of detectable antibodies (148). An association between the HLA-B*15:01 allele and silent SARS-CoV-2 infection has been suggested (149, 150). Preexisting cross-protective T cell–mediated immunity is a potential mechanism underlying abortive, seronegative SARS-CoV-2 infection (151), as well as silent infections (26, 149, 150). However, the CHGE failed to replicate any HLA allele association with asymptomatic SARS-CoV-2 infection in two large cohorts (149), in line with the modest impact of HLA variation on severe COVID-19 (26). In addition, robust T cell responses are found in both asymptomatic and symptomatic individuals (148), indicating that their presence *per se* does not explain silent infections. Thus, other mechanisms beyond T cells are likely to be involved. Monitoring of PCR-negative and seronegative healthcare workers has revealed preexisting cross-protective T cell–mediated immunity as a potential mechanism underlying abortive, seronegative SARS-CoV-2 infection (151). In addition to T cells, the contribution of innate immune responses to silent infections is poorly understood. Early type III IFN production can prevent viral replication and spread (152), and it has been suggested that early type I IFN production and NK cell activation can prevent the development of severe disease (153, 154, 155, 156). The host determinants underlying silent infections, including the possible contribution of innate immune responses, remain to be discovered.

Human genetic correlates of protection against infection have emerged from GWAS (26). A *cis*-expression quantitative trait locus in *ACE2* impacts infection susceptibility (OR ± 0.70) by reducing *ACE2* expression (26, 157). The O allele of the *ABO* locus has a small (OR ± 0.90) effect on susceptibility to infection, but the mechanism is unclear (26). For other protective alleles (26), the causal variants remain unknown. Testing the hypothesis that monogenic inborn variants confer natural resistance to SARS-CoV-2 infection, COVID HGE is analyzing >800 individuals who are long-term–resistant to SARS-CoV-2 infection (158). In line with the GWAS results (26), no associations of classical HLA alleles were found for resisters (149). Genome-wide analyses and molecular studies are ongoing. The proportion of humans naturally resistant to SARS-CoV-2 infection sensu stricto (being those individuals not undergoing abortive or silent infections) is unknown, and the human genetic determinants of resistance remain uncharted. Historical examples of inborn resistance to infection with other pathogens (e.g., HIV, norovirus, and *Plasmodium vivax*) (158) provide a road map for testing the hypothesis of monogenic resistance to infection with SARS-CoV-2. The genetic investigation of potential pan-coronavirus host resistance mechanisms (159) in individuals who are naturally resistant to SARS-CoV-2 infection deserves ongoing attention in the context of preparedness for future pandemics.

### 7. Mild and severe adverse reactions to RNA vaccines

Understanding the basic biology behind severe adverse reaction to the RNA vaccine emerged as a major enigma with public health implications. Clinical trials for COVID-19 vaccines reported interindividual differences for common side effects such as fever in the days following vaccination (160). Additionally, after hundreds of millions of people had been vaccinated worldwide, vaccination was conclusively linked to a few rare adverse events, particularly myocarditis following COVID-19 mRNA vaccination (161) and vaccine-induced immune thrombocytopenia and thrombosis (VITT) following the adenoviral-vector vaccines (162). Deciphering the genetic and immunological basis of adverse reactions to COVID-19 vaccines could provide insights into how the new type of mRNA vaccine works in humans. We first discovered that the HLA-A*03:01 allele was associated with fever, chills, and stronger side effects from the Pfizer-BioNTech mRNA vaccine (163). Later, HLA-A*03 individuals were found to have the highest increase in S-reactive CD8^+^ T cells following the second dose of vaccination due to enhanced antigen presentation (164). These results highlight how specific HLA alleles can modulate the response to mRNA vaccines. Possible HLA associations with vaccine-associated myocarditis were reported but were not statistically significant due to the low number of cases and controls (165). Epidemiological studies showed that vaccine-associated myocarditis affects about 1 in 100,000 vaccinees. It is more common in adolescent and young adult males, and after the second dose when the prevalence may reach 35 per 100,000 (166). Results from immune profiling of vaccine-associated myocarditis cases were consistent with a cytokine-mediated pathology, as opposed to an autoimmune myocarditis or a hypersensitivity reaction (167). However, why only a few individuals develop this abnormal immune response following vaccination remains unknown.

To uncover a potential genetic cause for vaccine-associated myocarditis (168), the CHGE has included and sequenced cases of vaccine-associated myocarditis if they meet the “Probable myocarditis” definition of the Brighton collaboration and are diagnosed within 2 wk of immunization. Comparing the genetic results from this ongoing study with cohorts of SARS-CoV-2 infection–associated myocarditis has the added potential to help pinpoint whether the immune trigger was the antigen (the spike protein), the adjuvant (the lipid nanoparticle), or the modified mRNA itself. While the scientific community has focused on myocarditis because it is the most common adverse event to mRNA vaccines, future work aims at understanding the cause of other rare adverse events to mRNA and other types of COVID-19 vaccines. It has been shown that VITT is mediated by platelet-activating antibodies against platelet factor 4 (169). However, why a few individuals have these antibodies remains unknown. GBS is another adverse event, whose pathogenic mechanism is not understood. Rather than focus on individual types of adverse effects, an alternative strategy would be to aim at all adverse events following vaccination in population-wide cohorts at once. This could be done by screening for a wide array of autoantibodies, as has been done in a recent Swedish study of all individuals with adverse events (170), combined with the search for rare genetic lesions affecting the same pathway. Future research to better understanding these vaccine reactions is of high importance and relevance if mRNA vaccines are to be applied to elicit protection against other viruses and in other contexts such as for cancer vaccines.

## Beyond the seven enigmas

While the initial effort of the CHGE focused on COVID-19 in unvaccinated people, and then expanded its effort to tackle other enigmas, it also studied “breakthrough” COVID-19 pneumonia in vaccinated people. Moreover, some discoveries appeared to be generalizable to other viruses. Indeed, the efforts to understand and prevent COVID-19 were fruitful beyond COVID-19, as neatly illustrated by the role of auto-Abs against type I IFN in other viral diseases and the development of RNA vaccines for other viral infections.

### 1. Life-threatening breakthrough COVID-19 pneumonia

While the seven aforementioned enigmas typically dealt with clinical phenotypes in unvaccinated individuals, RNA vaccines changed the course of the pandemic. “Breakthrough infection” is defined by SARS-CoV-2 infection occurring after a well-conducted COVID-19 vaccine series (171, 172). Most breakthrough cases are asymptomatic or mild (171), but in rare cases, they are severe, critical, or even fatal (173, 174). Breakthrough COVID-19 pneumonia is thematically related to COVID-19 pneumonia in unvaccinated individuals. As such, it cannot be seen as a distinctive enigma, although it deserves studies of its own. Breakthrough infection, regardless of its severity, can be of at least four causes: (1) primary or secondary vaccine failure (for example, in patients [including inherited and acquired deficiencies of adaptive immunity]), (2) waning antibody response to the vaccine (especially in aging individuals or those unable to sustain titers over time), (3) viral escape or viral resistance (leading to incomplete protection from viral genotypes with vaccine-resilient mutations [such as Delta or Omicron], which can result in insufficient viral neutralization in vivo), and (4) host susceptibility to severe infections that is so high that it overcomes a vaccine protection that is usually adequate (6). Patients with IEI affecting the production of or response to type I IFNs, or both, are prone to critical COVID-19 pneumonia (7, 175, 176). These latter findings established the crucial role of type I IFNs in fending off SARS-CoV-2 (6). Despite the efficacy of RNA vaccines (160, 177), the human genetic and immunological determinants of critical breakthrough cases remained unclear, especially in patients with normal antibody responses to the vaccine. With the CHGE (https://www.covidhge.com), we recruited and tested patients with breakthrough hypoxemic COVID-19 pneumonia. We hypothesized that some of these breakthrough cases of life-threatening COVID-19 pneumonia might have an adequate antibody response to the vaccine yet may harbor auto-Abs to type I IFNs leading to severe infection.

We thus tested 42 individuals with no known deficiency of B cell immunity and normal antibody responses to two doses of an mRNA vaccine and found that 10 (24%) patients (aged 43–86 years) had auto-Abs neutralizing type I IFNs (178). For most patients with type I IFN defects (either genetic or autoimmune), protection conferred by COVID-19 mRNA vaccination is probably sufficient, yet additional boosters might be needed in some individuals who neutralize high concentrations of multiple type I IFNs. We know of no other reported risk factor that leads to severe breakthrough infection in individuals who mount adequate Ab responses. Specific vaccination or preventive strategies could be undertaken in such at-risk individuals, such as early treatments with antivirals and/or IFNs (179, 180, 181, 182), or additional vaccine boosters.

### 2. Auto-Abs against type I IFNs

Auto-Abs neutralizing type I IFNs were first described in 1981 in a patient previously treated with human IFN-β (183). Soon after, auto-Abs neutralizing IFN-α were detected in a patient with disseminated varicella zoster disease prior to any treatment with type I IFNs (184, 185). Nevertheless, for almost 40 years these auto-Abs were largely thought to be uncommon, induced by type I IFN treatment or restricted to rare conditions, and clinically silent with respect to viral infections (186, 187, 188). During the pandemic, the study of cohorts of thousands of patients facilitated the identification in ∼20% of COVID-19 pneumonia fatal cases of preexisting circulating auto-Abs neutralizing the 12 types of IFN-α (encoded by 13 loci) and/or IFN-ω and/or, less frequently, IFN-β (6, 21, 22, 23, 24, 178) (Table 1). This finding has been replicated in >30 independent cohorts worldwide (23, 24, 189, 190, 191, 192, 193, 194, 195, 196, 197, 198, 199, 200, 201, 202, 203, 204, 205, 206, 207, 208, 209, 210, 211, 212, 213, 214, 215, 216, 217, 218, 219, 220, 221) and, later, in ∼10% of cases of pediatric hospitalizations for COVID-19 pneumonia (24). Interestingly, there is a male bias in individuals harboring such auto-Abs, especially in the elderly (21, 22, 23). These discoveries were soon followed by the identification of the auto-Abs in ∼5% of cases of critical influenza pneumonia (41) and ∼25% of hospitalizations for Middle East respiratory syndrome (45), two respiratory diseases with pandemic potential caused by RNA viruses, and in patients with severe disease caused by five flaviviruses, including ∼35% of cases of life-threatening adverse reactions to yellow fever live-attenuated vaccine-17D strain (44, 222), ∼40% of cases of WNV encephalitis (38), ∼10% of severe cases of tick-borne encephalitis virus disease (TBEV) (37), and severe Powassan virus (POWV) and Usutu virus (USUV) disease, as well as severe disease caused by Ross River virus (RRV), an alphavirus (223) (Table 1). These auto-Abs also increase the risk of skin infection by HSV-1 and VZV (189, 224, 225, 226). Conversely, in a cohort of >35,000 unselected individuals aged 0–90 years from the general population, they were found to be uncommon under the age of 65 years (prevalence of ∼0.3–1%), with a sharp increase in their prevalence after the age of 70 years (∼4–7%), particularly in men, a finding that can explain part of the age- and sex-dependent risk of life-threatening COVID-19 (6, 22, 23).

**Table 1.** Infections explained by auto-Abs against type I IFN and OR as compared to the general population

Infections	% of cases with auto-Abs	OR (95% confidence interval)	
		IFN-α and/or IFN-ω (100 pg/ml)	IFN-α and IFN-ω (10 ng/ml)
Critical COVID-19 pneumonia	14%	13 (8–21)	67 (4–1,109)
Fatal COVID-19 under 70 years old	21%	17.0 (11.7–24.8)	156.5 (57.8–423.4)
Fatal COVID-19 above 70 years old	15%	5.8 (4.5–7.4)	12.9 (8.4–19.9)
Pediatric COVID-19 pneumonia	10%	5.3 (2.8–9.6)	112 (12–14,991)
Breakthrough hypoxemic COVID-19 pneumonia	24%	-	-
Severe flu before 70 years old	6%	5.7 (3.0–11.1)	139.9 (42.3–462.5)
Avian flu	100%^a^	-	-
WNV neuroinvasive disease (study 1)	39%	21.1 (16.4–27.1)	138.4 (93.3–205.4)
Under 65 years old	22%	24.4 (14.4–40.4)	702 (266–2,149)
Above 65 years old	47%	24.5 (18.0–33.4)	85.8 (56.8–131.2)
WNV neuroinvasive disease (study 2)	38%	16.8 (12.0–23.3)	101.3 (63.2–162.3)
Under 65 years old	23%	26.6 (17.4–40.7)	602 (224.2–1,616.2)
Above 65 years old	44%	22.3 (17.1–29.1)	84.6 (57.6–124.2)
Severe TBEV encephalitis	10%	4.9 (1.5–15.9)	20.8 (4.5–97.4)
Severe POWV encephalitis	100%^a^	-	-
Severe USUV infection	67%	-	-
Severe RRV disease	4%^b^	-	-
Yellow fever virus vaccine disease	38%	-	-
Severe reaction to chikungunya vaccine	60%	-	-
HSV-triggered fulminant viral hepatitis	38%	29.7 (11.3–77.9)	1,873.9 (444.4–7,901.8)

a *n* = 1 patient.

b Auto-abs against type I IFNs carried by the most severe patient out of 24 defined as severe.

These auto-Abs neutralize the antiviral activity of type I IFNs in vitro (21, 37, 38, 41, 44) by preventing the induction of ISGs (22, 199, 227), thus clinically phenocopying inborn errors of type I IFN immunity due to autosomal recessive IFNAR1 or IFNAR2 deficiency (6, 70, 228). The auto-Abs confer increased risk whose magnitude depends on the number and concentrations of type I IFNs neutralized (22). The auto-Abs have also been found in the bronchoalveolar lavage fluid of patients with life-threatening COVID-19 pneumonia (218), and in the cerebrospinal fluid of patients with WNV encephalitis (38), where they probably contribute to organ-specific viral disease. Recently, the characterization of repertoire, clonal maturation, and antibody diversity of circulating type I IFN–specific B cells from patients with life-threatening COVID-19 pneumonia demonstrated that highly mutated memory B cells producing high-affinity auto-Abs had undergone extensive T cell–dependent germinal center maturation prior to SARS-CoV-2 infection, establishing that they preexisted COVID-19 pneumonia in these patients (229). These findings also point to defective thymic T cell tolerance, rather than defective B cell tolerance, as the underlying immunological defect for germinal center maturation of germline autoreactive B cell clones (230).

Consistently, auto-Abs neutralizing type I IFNs have been found in a growing number of inborn errors of thymic tolerance, due to T cell–intrinsic impairment of thymocyte maturation in patients with autosomal recessive (AR) partial RAG1 or RAG2 deficiency (231) and in about one third of patients with X-linked recessive FOXP3 deficiency (232), or to impaired development of *AIRE*-expressing medullary thymic epithelial cells in most patients with autoimmune polyendocrine syndrome type 1 (APS-1) (187, 192, 226, 233, 234, 235), inborn errors of the alternative NF-κB pathway (224, 225, 236, 237), and incontinentia pigmenti (238), as well as in patients with loss-of-function mutations in *IKZF2* (239) and one patient with pre-TCR-α deficiency (240). In the subsequent years, auto-Abs neutralizing type I IFNs were found in patients and cohorts with hepatitis C virus disease (241, 242, 243, 244), multiple sclerosis (245), myasthenia gravis and thymoma (246, 247), and systemic lupus erythematosus (186, 248, 249) following or not prior treatment with IFN-α or IFN-β. More recently, auto-Abs neutralizing type I IFNs were found in rare single-gene disorders, including APS-1 (187, 233, 234), RAG1 and RAG2 deficiency (231), and immune dysregulation, polyendocrinopathy, enteropathy, X-linked (232).

Overall, following the identification in COVID-19 pneumonia in 2020, auto-Abs neutralizing IFN-α and/or IFN-β and/or IFN-ω emerged as universal determinants of susceptibility to viral disease, underlying a growing number of viral infections, regardless of the mechanism underlying their generation (Table 1). Different approaches are being developed for the detection of auto-Abs neutralizing type I IFNs in clinical and research settings (41, 250), including a simple and fast whole blood assay that can be used for rapid diagnosis of rare inborn errors of the type I IFN response pathway and the more common auto-Abs neutralizing type I IFNs (251). These assays can screen for at-risk individuals and populations, as well as patients in the course of viral infection. The detection of these auto-Abs is key to prevention, currently possible through measures to reduce exposure and vaccination, and for treatment stratification and prioritization for antivirals and targeted drugs, including antiviral monoclonal Abs (181, 252). While clinical trials have been hampered by late administration of type I IFNs, which may lack efficacy and may even be deleterious (253), one trial in COVID-19 patients showed benefit from type I IFN administration during the early course of infection (253, 254). Furthermore, a trial of early IFN-β administration for WNV encephalitis with stratification according to carriage of auto-Abs neutralizing type I IFNs is ongoing (255), overall suggesting the continued broad medical relevance of detecting these auto-Abs.

### 3. RNA vaccines in the context of type I IFN deficiency

The three clinical pillars of vaccinology are efficacy, safety, and effectiveness. Over a decade’s worth of research in the development of mRNA vaccines showed that, when left in their native configuration, they were marred by inherent instability and low level of protein expression due to their high innate immunogenicity. Specifically, foreign mRNA (whether from infecting microbe or inoculated vaccine) is recognized by various innate immune sensors to induce type I IFN and other proinflammatory cytokines to eliminate the foreign mRNA and/or the cells that harbor them. Their recognition by innate immune sensing pathways can be prevented by nucleoside modification (i.e., the integration of N1-methylpseudouridine [m1Ψ] in the mRNA structure), which results in enhanced stability and translational efficiency (256, 257). Lipid nanoparticles were also developed to optimize nucleic acid delivery. This platform was rapidly mobilized to tackle the SARS-CoV-2 pandemic, with the original and subsequent iterations of mRNA vaccines demonstrating excellent efficacy (variant-specific seroconversion), safety profiles (through global monitoring of adverse events following immunization [AEFI]), and real-world effectiveness (258, 259, 260, 261).

Because deficient type I IFN responses (through monogenic or autoantibody-mediated processes) underlie life-threatening COVID-19, there is particular interest in whether such affected individuals may have compromised or untoward reactions to the mRNA vaccines. From the perspective of putative compromised efficacy, type I IFNs are involved not only in innate inhibition of viral replication, but also were shown to induce adaptive immunity (262). Theoretically, humans with deficient type I IFN immunity may have suboptimal or even absent vaccine responses, rendering them at persistent risk for severe disease. However, such concerns did not manifest. Patients with monogenic defects in either the production or response to type I IFN (*TLR7*, *IRF7*, *IFNAR1*), including those with monogenic predisposition to producing autoantibodies against type I IFNs (mutations in *AIRE*), and older adults with age-associated autoantibodies to type I IFNs, had humoral response to mRNA vaccination that was similar in titer and duration to healthy controls, as were germinal center responses (263). However, since that study was conducted at a time when two doses of the original vaccine were recommended, and because autoantibodies to type I IFN were found to underlie breakthrough COVID-19 (178), such individuals were recommended to receive a third dose of vaccine, similar to other immunocompromised populations. In practicality, such individuals should probably continue to receive the currently updated SARS-CoV-2–formulated vaccine, as well as the influenza vaccines. Similarly, using the Swedish national registry for AEFI, no association between autoantibodies to type I IFN and any of the most commonly reported AEFI was found (170). Due to the nature of the study, and the rarity of monogenic defects in type I IFN, the study could not specifically analyze AEFI in the latter group of patients. However, it should be noted that the severe AEFI in those with genetic deficiency of type I IFN responses have occurred to live-attenuated vaccines (43, 44, 264, 265), suggesting that the mRNA or protein subunit vaccines may not pose a heightened risk for adverse events in these individuals. Collectively, the data reinforce the importance of the mRNA vaccines in effectively and safely protecting patients, including those with type I IFN deficiency.

## Conclusion

The strength of the CHGE derives from a global group of collaborators directly or indirectly studying IEI or other approaches to the human genetics of infectious diseases, a field in which global collaboration has been the origin of numerous discoveries (266, 267). From a small group, the consortium grew to include more teams. The CHGE held weekly meetings to share insights, hypotheses, and questions. The breakthroughs we made were based on sequential studies of rare patients with outlier phenotypes. Importantly, our initial insights were not restricted to rare patients, but were later expanded to larger cohorts of patients, namely, those lacking genetic defects but having phenocopies due to autoantibodies, and those with other types of viral infections besides COVID-19. The overarching lesson is that global cooperation to study rare and even single patients can unlock a problem that is more common with profound implications and have broader relevance, in terms of basic biology and public health. This is the road we will follow to tackle unexplained cases of critical COVID-19, MIS-C, and neuro-COVID, as well as other enigmas, including the COVID-toes, long COVID, resistance/silent infections, and adverse reactions to vaccine. We expect that our continued efforts will also provide broad insights that are essential for mitigating the impact of future pandemics, whether to new coronaviruses or other emerging viruses. The application of this “model” to future emerging pathogens is likely to reap the same clinical benefits while also enlightening mechanisms of protective immunity and infectious disease.

## Supplementary Material

Table S1 lists the COVID Human Genetic Effort members and affiliations.
