# Supplementary material for: The seven enigmas of SARS-CoV-2: From the past to the future
Source: J Hum Immun. 2025 Oct 24;1(4):e20250149. doi: 10.70962/jhi.20250149 (PMC12674185; doi:10.70962/jhi.20250149)
Supplement: Table S1 — lists the COVID Human Genetic Effort members and affiliations. [file jhi_20250149_tables1.docx]

**COVID Human Genetic Effort:**

Laurent Abel^1^, Alessandro Aiuti^2^, Saleh Al-Muhsen^3^, Evangelos Andreakos^4^, Andrés A. Arias^5^, Lisa M. Arkin^6^, Hagit Baris Feldman^7^, Paul Bastard^1^, Alexandre Bolze^8^, Anastasiia Bondarenko^9^, Alessandro Borghesi^10^, Ahmed A. Bousfiha^11^, Petter Brodin^12^, Giorgio Casari^13^, John Christodoulou^14^, Aurélie Cobat^1^, Roger Colobran^15^, Antonio Condino-Neto^16^, Stefan N. Constantinescu^17^, Beth A. Drolet^18^, Munis Dündar^19^, Sara Espinosa-Padilla^20^, Jacques Fellay^21^, Carlos Flores^22^, Antoine Froidure^23^, Guy Gorochov^24^, David Hagin^25^, Rabih Halwani^26^, Lennart Hammarström^27^, Elena W. Y. Hsieh^28^, Yuval Itan^29^, Emmanuelle Jouanguy^1^, Elżbieta Kaja^30^, Yu-Lung Lau^31^, Davood Mansouri^32^, László Maródi^33^, Isabelle Meyts^34^, Trine H. Mogensen^35^, Lisa F.P. Ng^36^, Antonio Novelli^37^, Giuseppe Novelli^38^, Satoshi Okada^39^, Keisuke Okamoto^40^, Firat Ozcelik^41^, Qiang Pan-Hammarström^27^, Rebeca Perez de Diego^42^, David S. Perlin^43^, Anne Puel^1^, Aurora Pujol^44^, Laurent Renia^36^, Vanessa Sancho-Shimizu^45^, Mohammad Shahrooei^46^, Anna Shcherbina^47^, Ondrej Slaby^48^, Pere Soler-Palacín^49^, András N. Spaan^50^, Ivan Tancevski^51^, Stuart G. Tangye^52^, Ahmad Abou Tayoun^53^, Christian Thorball^54^, Pierre Tiberghien^55^, Stuart E. Turvey^56^, Donald C. Vinh^57^, Qian Zhang^1^, Shen-Ying Zhang^1^, Helen C. Su^58^, Jean-Laurent Casanova^59^

^1^Laboratory of Human Genetics of Infectious Diseases, Necker Branch, INSERM U1163, Necker Hospital for Sick Children, Paris, France; Paris Cité University, Imagine Institute, Paris, France; St. Giles Laboratory of Human Genetics of Infectious Diseases, Rockefeller Branch, Rockefeller University, New York, NY, USA.

^2^San Raffaele Telethon Institute for Gene Therapy, IRCCS Ospedale San Raffaele, and Vita Salute San Raffaele University, Milan, Italy.

^3^Immunology Research Lab, Department of Pediatrics, College of Medicine, King Saud University, Riyadh, Saudi Arabia.

^4^Laboratory of Immunobiology, Center for Clinical, Experimental Surgery and Translational Research, Biomedical Research Foundation of the Academy of Athens, Athens, Greece.

^5^St. Giles Laboratory of Human Genetics of Infectious Diseases, Rockefeller Branch, The Rockefeller University, New York, NY, USA; Primary Immunodeficiencies Group, Department of Microbiology and Parasitology, School of Medicine, University of Antioquia, Medellín, Colombia; School of Microbiology, University of Antioquia UdeA, Medellín, Colombia.

^6^Department of Dermatology, School of Medicine and Public Health, University of Wisconsin-Madison, Madison, WI, USA.

^7^The Genetics Institute, Tel Aviv Sourasky Medical Center and Sackler Faculty of Medicine, Tel Aviv University, Tel Aviv, Israel.

^8^Helix, San Mateo, CA, USA.

^9^International European University, Kyiv, Ukraine.

^10^School of Life Sciences, Swiss Federal Institute of Technology , Lausanne, Switzerland; Neonatal Intensive Care Unit, San Matteo Research Hospital , Pavia, Italy.

^11^Clinical Immunology and Infectious Pediatrics Department, Abderrahim Harouchi Hospital-Ibn Rochd University Hospital; Laboratory of Clinical Immunology-Inflammation and Allergy (LICIA), Faculty of Medicine and Pharmacy, Hassan II University, Casablanca, Morocco.

^12^SciLifeLab, Department of Women’s and Children’s Health, Karolinska Institutet, Stockholm, Sweden.

^13^Clinical Genomics, IRCCS San Raffaele Scientific Institute and Vita-Salute San Raffaele University, Milan, Italy.

^14^Murdoch Children’s Research Institute and Department of Paediatrics, University of Melbourne, Melbourne, VIC, Australia.

^15^Immunology Division, Genetics Department, Hospital Universitari Vall d’Hebron, Vall d’Hebron Research Institute, Vall d’Hebron Barcelona Hospital Campus, UAB, Barcelona, Spain.

^16^Department of Immunology, Institute of Biomedical Sciences, University of São Paulo, São Paulo, Brazil.

^17^de Duve Institute and Ludwig Cancer Research, Brussels, Belgium.

^18^School of Medicine and Public Health, University of Wisconsin, Madison, WI, USA.

^19^Department of Medical Genetics, Faculty of Medicine, Erciyes University, Kayseri, Turkey.

^20^Immune Deficiencies Laboratory, National Institute of Pediatrics, Mexico City, Mexico.

^21^School of Life Sciences, Ecole Polytechnique Fédérale de Lausanne, Lausanne, Switzerland; Precision Medicine Unit, Lausanne University Hospital and University of Lausanne, Lausanne, Switzerland.

^22^Research Unit, Hospital Universitario Nuestra Señora de Candelaria, Santa Cruz de Tenerife; CIBER de Enfermedades Respiratorias, Instituto de Salud Carlos III, Madrid; Genomics Division, Instituto Tecnológico y de Energías Renovables (ITER), Santa Cruz de Tenerife, Spain; Faculty of Health Sciences, University of Fernando Pessoa Canarias, Las Palmas de Gran Canaria, Spain.

^23^Pulmonology Department, Cliniques Universitaires Saint-Luc ; Institut de Recherche Expérimentale et Clinique (IREC), Université Catholique de Louvain, Brussels, Belgium.

^24^Sorbonne Université, Inserm, Centre d’Immunologie et des Maladies Infectieuses-Paris (CIMI PARIS), Assistance Publique-Hôpitaux de Paris (AP-HP) Hôpital Pitié-Salpêtrière, Paris, France.

^25^The Genetics Institute Tel Aviv Sourasky Medical Center, Tel Aviv, Israel.

^26^Sharjah Institute of Medical Research, College of Medicine, University of Sharjah, Sharjah, United Arab Emirates.

^27^Department of Biosciences and Nutrition, Karolinska Institutet, Stockholm, Sweden.

^28^Departments of Pediatrics, Immunology and Microbiology, University of Colorado, School of Medicine, Aurora, CO, USA.

^29^Institute for Personalized Medicine, Icahn School of Medicine at Mount Sinai, New York, NY, USA; Department of Genetics and Genomic Sciences, Icahn School of Medicine at Mount Sinai, New York, NY, USA.

^30^Department of Medical Chemistry and Laboratory Medicine, Poznan University of Medical Sciences, Poznan, Poland.

^31^Department of Paediatrics & Adolescent Medicine, The University of Hong Kong, Hong Kong, China.

^32^Department of Clinical Immunology and Infectious Diseases, National Research Institute of Tuberculosis and Lung Diseases, The Clinical Tuberculosis and Epidemiology Research Center, National Research Institute of Tuberculosis and Lung Diseases (NRITLD), Masih Daneshvari Hospital, Shahid Beheshti, University of Medical Sciences, Tehran, Iran.

^33^Primary Immunodeficiency Clinical Unit and Laboratory, Department of Dermatology, Venereology and Dermatooncology, Semmelweis University, Budapest, Hungary.

^34^Department of Pediatrics, University Hospitals Leuven; KU Leuven, Department of Microbiology, Immunology and Transplantation; Laboratory for Inborn Errors of Immunity, KU Leuven, Leuven, Belgium.

^35^Department of Biomedicine, Aarhus University, Aarhus, Denmark.

^36^A*STAR Infectious Disease Labs, Agency for Science, Technology and Research, Singapore; Lee Kong Chian School of Medicine, Nanyang Technology University, Singapore.

^37^Laboratory of Medical Genetics, IRCCS Bambino Gesù Children’s Hospital, Rome, Italy.

^38^Department of Biomedicine and Prevention, Tor Vergata University of Rome, Rome, Italy.

^39^Department of Pediatrics, Graduate School of Biomedical and Health Sciences, Hiroshima University, Hiroshima, Japan.

^40^Tokyo Medical and Dental University, Tokyo, Japan.

^41^Department of Medical Genetics, School of Medicine, Erciyes University, Kayseri, Turkey, ^42^Institute of Biomedical Research of IdiPAZ, University Hospital “La Paz”, Madrid, Spain.

^43^Center for Discovery and Innovation, Hackensack Meridian Health, Nutley, NJ, USA.

^44^Neurometabolic Diseases Laboratory, Institut d’Investigació Biomèdica de Bellvitge (IDIBELL), Hospital Duran i Reynals, Barcelona, Spain; Center for Biomedical Research on Rare Diseases, (CIBERER U759) Ministry of Science Innovation and University, Madrid, Spain; Catalan Institution of Research and Advanced Studies (ICREA), Barcelona, Spain.

^45^Department of Paediatric Infectious Diseases and Virology, Imperial College London, London, UK; Centre for Paediatrics and Child Health, Faculty of Medicine, Imperial College London, London, UK.

^46^Dr. Shahrooei Lab, Tehran, Iran.

^47^Department of Immunology, Dmitry Rogachev National Medical Research Center of Pediatric Hematology, Oncology and Immunology, Moscow, Russia.

^48^Central European Institute of Technology & Department of Biology, Faculty of Medicine, Masaryk University, Brno, Czech Republic.

^49^Pediatric Infectious Diseases and Immunodeficiencies Unit, Vall d’Hebron Barcelona Hospital Campus, Barcelona, Catalonia, Spain.

^50^St. Giles Laboratory of Human Genetics of Infectious Diseases, Rockefeller Branch, The Rockefeller University, New York, NY, USA; Department of Medical Microbiology, University Medical Center Utrecht, Utrecht, Netherlands.

^51^Department of Internal Medicine II, Medical University of Innsbruck, Innsbruck, Austria.

^52^Garvan Institute of Medical Research, Darlinghurst, NSW, Australia; St Vincent’s Clinical School, Faculty of Medicine, UNSW Sydney, NSW, Australia.

^53^Al Jalila Children’s Hospital, Dubai, UAE.

^54^Precision Medicine Unit, Lausanne University Hospital and University of Lausanne, Lausanne, Switzerland.

^55^Etablissement Francais Du Sang, La Plaine-Saint Denis, Saint-Denis, France.

^56^BC Children’s Hospital, The University of British Columbia, Vancouver, Canada.

^57^Department of Medicine, Division of Infectious Diseases, McGill University Health Centre, Montréal, Québec, Canada; Infectious Disease Susceptibility Program, Research Institute, McGill University Health Centre, Montréal, Québec, Canada.

^58^National Institute of Allergy and Infectious Diseases, National Institutes of Health, Bethesda, MD, USA.

^59^The Rockefeller University & Howard Hughes Medical Institute, New York, NY, USA; Necker Hospital for Sick Children & INSERM, Paris, France.
